# Assessing the Ability to Use eHealth Resources Among Older Adults: Cross-Sectional Survey Study

**DOI:** 10.2196/70672

**Published:** 2025-08-06

**Authors:** Bernard Aoun, Jon O Ebbert, Priya Ramar, Daniel L Roellinger, Lindsey M Philpot

**Affiliations:** 1 Division of Community Internal Medicine Geriatrics and Palliative Care, Department of Quantitative Health Sciences, Mayo Clinic, 200 First Street SW, Rochester, MN, 55905, United States

**Keywords:** patient portal, digital health, digital literacy, older adults, socioeconomic deprivation

## Abstract

**Background:**

Increasing reliance on digital health resources can create disparities among older patients. Understanding health-related, mobility, and socioeconomic factors associated with the use of eHealth technologies is important for addressing inequitable access to health care.

**Objective:**

We sought to assess digital health literacy among patients aged ≥65 years and identify factors associated with their ability to access, understand, and use digital health resources.

**Methods:**

We developed a survey instrument grounded in the Technology Acceptance Model and conducted a cross-sectional, mixed-mode survey of patients aged ≥65 years from an integrated, multispecialty medical center. Digital health literacy was measured using the eHeals health literacy scale, and responses were analyzed across self-rated health, self-reported mobility, and socioeconomic deprivation assessed with the Area Deprivation Index (ADI). Counts (n) and frequencies (%) are reported across response groups, and analyses for differences are performed using the *χ*^2^ test for independence or the Fisher exact test.

**Results:**

Analyses included 878 responses (response rate=878/2847; 30.8%). There was a significant difference in the distribution of race between responders and nonresponders (*P*<.001) but no significant differences were observed by age (*P*=.053) or gender (*P*=.73). Respondents with lower self-rated health had lower levels of digital health literacy; only 54.2% (n=13/25) participants with poor self-rated health were able to send a message to their doctor compared to 89.5% (n=68/77) of patients with excellent self-rated health. All comparisons across the digital health literacy domains revealed significant differences across self-rated health groups (*P*<.05). Respondents with mobility restrictions had lower levels of digital health literacy, including lower frequencies of reporting knowledge of what health resources are available on the internet (mobility restricted, n=92/182; 52.0% vs no mobility restriction, n=433/688; 64.7%), knowledge of how to find health resources on the internet (mobility restricted, n=120/182; 67.4% vs no mobility restriction, n=513/688; 76.8%), and ability to use a camera or video with a doctor easily (mobility restricted, n=58/182; 32.6% vs no mobility restriction, n=321/688; 48.0%). Older adults experiencing increased socioeconomic deprivation, as measured by the ADI, reported lower rates of digital health literacy across most categories, including knowledge of how to find health resources on the internet (high ADI, n=28/49; 59.6% vs low ADI, n=551/751; 75.5%) and the ability to send an electronic message to their doctor easily (high ADI, n=27/49; 57.4% vs low ADI, n=584/751; 80.2%).

**Conclusions:**

Our findings highlight the need for targeted interventions to improve engagement with eHealth among patients aged ≥65 years, who are impacted by poor health, limited mobility, and socioeconomic deprivation. Enhancing digital health literacy can help bridge the gap in access to digital health resources and improve overall health outcomes for this population.

## Introduction

Digital health care (eHealth) has become a fundamental feature of modern clinical practice. eHealth encompasses a range of digital tools that support health care delivery and patient-provider communication. This includes patient portals, telehealth services, mobile apps, and online health resources that are increasingly integrated into everyday care. Its adoption has accelerated during the COVID-19 pandemic [1] with the development of dependable platforms, health care workforce shortages, and increases in demand for health care services in growing urban and rural areas. Digital health literacy is essential for patients to use online health tools effectively. It includes the ability to find, interpret, and apply electronic health information, as well as skills for participating in telehealth visits and communicating with providers.

Older adults are expected to constitute a larger share of the population [2], and they face barriers to digital engagement due to changes in health, mobility, and finances placing them at a disadvantage with the growing introduction of digital health modalities. Internet usage decreases with advancing age, especially among those lacking a college education, which is further constrained by chronic health conditions and health care access [3]. In addition, patient health status changes, such as the onset of cognitive impairment, placement in a long-term care facility, or declining physical capabilities can compromise their ability to use technological solutions [4]. Higher education levels, autonomous internet use, possession of internet-capable devices, and earlier engagement with patient portal messaging increase the probability of technology adoption [5].

Investigations of factors influencing the adoption of eHealth by seniors have highlighted the impact of age, educational level, socioeconomic status, health literacy, and ability to engage with digital resources [6,7]. Higher levels of socioeconomic deprivation, measured by the Area Deprivation Index (ADI), are consistently associated with reduced eHealth technology adoption, particularly telemedicine (eg, reliance on audio-only over video) [8-11], but extant research is limited in the examination of US-based older adult residents, ADI-specific subgroups, and contemporary data since the end of the COVID-19 pandemic. Patients with mobility limitations have been observed to use the internet for health-related tasks [12], which may have increased during the COVID-19 pandemic [13], but gaps may still remain. Direct links between self-reported health status and eHealth disparities are also underexamined, with most studies addressing health status via proxy measures (ie, comorbidities) rather than subjective health ratings [14-16].

In the current study, we sought to advance our understanding of the relationship between self-reported health, mobility, and socioeconomic deprivation and digital health literacy, specifically the confidence, skills, and use of eHealth resources. We analyzed data from a survey including 871 individuals aged ≥65 years responding to a questionnaire about digital health literacy. Moreover, we aimed to identify potential barriers to eHealth engagement in older adults that could be addressed through targeted interventions by policymakers and health care systems.

## Methods

### Study Overview

As the current investigation is a subanalysis of a larger study, full details of the study approach can be found in the original article [17]. In brief, a cross-sectional survey was developed and deployed using 2 modalities based on patient-indicated preference for contact: electronic deployment via Qualtrics Survey Software (Provo) to patient-provided e-mail address or paper survey delivered via US mail to patient-provided permanent residence. Stamped return envelopes were included with all paper-based, mailed surveys. Responses to both survey modalities were appended into a single dataset for analysis purposes. The Mayo Clinic Survey Research Center performed the build, deployment, and management of both survey modalities. The survey was deployed from August to October of 2023. This study investigated our research questions among the respondents aged ≥65 years at the time of survey completion.

### Study Population

As part of the routine process for clinical care delivery at any Mayo Clinic practice site, patients are offered enrollment within an electronic medical record-based patient portal. Within this portal, patients can access their medical chart information, view upcoming medical appointments, and send and receive messages from their medical team, among other features. At our institution, most patients register to use the patient portal (90% in 2022), while approximately 10% of patient accounts are inactive due to infrequent recent use, pending activation by the end user, or the patient declined enrollment. We sampled patients in a 2:1 ratio of nonregistered patients to registered and active patients to gain representation across both populations. Patients were included within the original sample if they were 18 years and older at the time of survey sampling, were still living according to the US Census and our internal medical records, and had selected English as their primary language within our medical record system. We sampled a total of 11,424 individuals for our primary analysis, of which 1850 (16.2%) completed our survey instrument and 871 were aged ≥65 years and included within the present secondary analysis.

### Survey Instrument

Based on the technology acceptance model, a widely used framework that explains how users come to accept and use a technology, an interdisciplinary team developed a survey instrument containing a mix of validated survey instruments and with novel survey items focusing on assessment of patient perceptions on the usefulness of an electronic medical record-based patient portal, patient perceptions on the ease of use of the patient portal, patient intent to use the patient portal, and actual use behavior of the portal. In addition, we incorporated a validated measure of digital health literacy. Face validation of the survey instrument was performed through 3 rounds of iterative testing and rewording. A copy of the complete deployed survey instrument is provided in Multimedia Appendix 1.

For this secondary analysis, a variety of demographic and technological ability assessments were used, including the eHEALS health literacy scale (an 8-item measure that assesses an individual’s knowledge, comfort, and perceived skills at finding, evaluating, and applying electronic health information to health problems), developed by Norman and Skinner [18]. Demographic information included age at the time of survey deployment (years and extracted from the medical record), gender, and race. Respondents were also asked to rate their level of comfort reading and speaking English, their ability to connect to the internet for general purposes, their ability to access connection devices (smartphone, laptop, personal computer, tablet, other), their ability to access the internet from home, and satisfaction with their internet connection from home.

To address our study aims, we asked respondents to indicate their current level of self-rated health on a 5-point Likert scale using the single item deployed within the National Health and Nutrition Examination Survey. This single-item report of self-rated health has demonstrated a correlation with the multidimensions of health and well-being, including social, economic, physical, environmental, and mental aspects of one’s life [19]. Reliability measures of this single-item self-rated health have been moderate to strong within large, population-based samples in the United States [20]. Answers reflect an individual’s comprehensive or holistic belief of their own well-being, and unlike objective clinical measures which require medical record access or clinical assessment, self-rated health can be easily and reliably collected. Furthermore, how people perceive their health may influence whether they see value in digital health resources, potentially being a more direct factor in technology adoption than objective disease measurements. To assess respondents' self-reported mobility, we used the mobility question from the EuroQual-5 dimension (EQ-5D) scale [21] (“I have no problems in walking about” versus “I have some problems in walking about” and “I am confined to bed” combined). The overall performance of the EQ-5D has been well documented among older adults, including validation and reliability [22,23]. To assess the relationship between socioeconomic deprivation (SED) and digital disparities, we deployed the standardized ADI [24]. ADI is a neighborhood-level approximation for SED based on a census block of 17 individual indicators, including median family income, percent of single-family households, and population with less than a high school education, among others. High scores of SED measured by the ADI correlate with poorer health outcomes, including higher rates of chronic disease associated with limited access to health care services, healthy food options, and safe spaces for recreation, shorter life expectancy due to higher exposure to environmental hazards and increased stress levels, and higher rates of mental health issues [25]. The reliability and validity of the ADI have been previously reported [26]. We created a dichotomized SED flag at the 80% standardized ADI rank value, as has been published elsewhere [27].

### Survey Fielding

Our cross-sectional survey was deployed via two modes to address the study aims, an electronic survey and a paper survey instrument. Contact information for the sampled population was derived from the institution’s integrated electronic medical record system. Deployment mode was selected based on patient-provided preferences for communications documented in the electronic medical record system. Electronic surveys were designed, managed, and deployed using Qualtrics survey software (Provo; Qualtrics) and included 3 total invitations to complete the survey instrument. Paper surveys were created using InDesign software (Microsoft Corporation) and deployed in a scannable booklet format via the US Postal Service in a single distribution wave. Stamped return envelopes were included with all mailed surveys. Those who did not respond to their electronic survey received a paper survey within 90 days of nonresponse. Responses to electronic and paper surveys were appended into a single dataset for analysis purposes. The build, deployment, and management of survey instruments were performed by the Mayo Clinic Survey Research Center.

### Statistical Analysis

Simple count (n) and proportion (%) are provided as descriptive statistics for all reported data. Comparisons across groups are performed either using the *χ*^2^ test for independence or the Fisher exact test when the assumptions of the *χ*^2^ test were not met. All data management and statistical analyses were performed using Statistical Analysis Software (SAS, version 9.2). Results were considered statistically significant if *P*<.05. Assessment for bias due to nonresponse is included in Multimedia Appendix 2.

### Ethical Considerations

This study (IRB#22‐008356; principal investigator: LM Philpot) underwent expedited review by the Mayo Clinic Institutional Review Board (Rochester, MN, USA) and was approved as exempt (45 CFR 46.104d, Category 2). As protected health information was not being requested from survey respondents, HIPAA (Health Insurance Portability and Accountability Act) authorization was not required in accordance with 45 CFR 160.103. Study data were anonymized, deidentified, and only presented in aggregate form. No compensation was provided to survey respondents.

## Results

**Figure 1.**
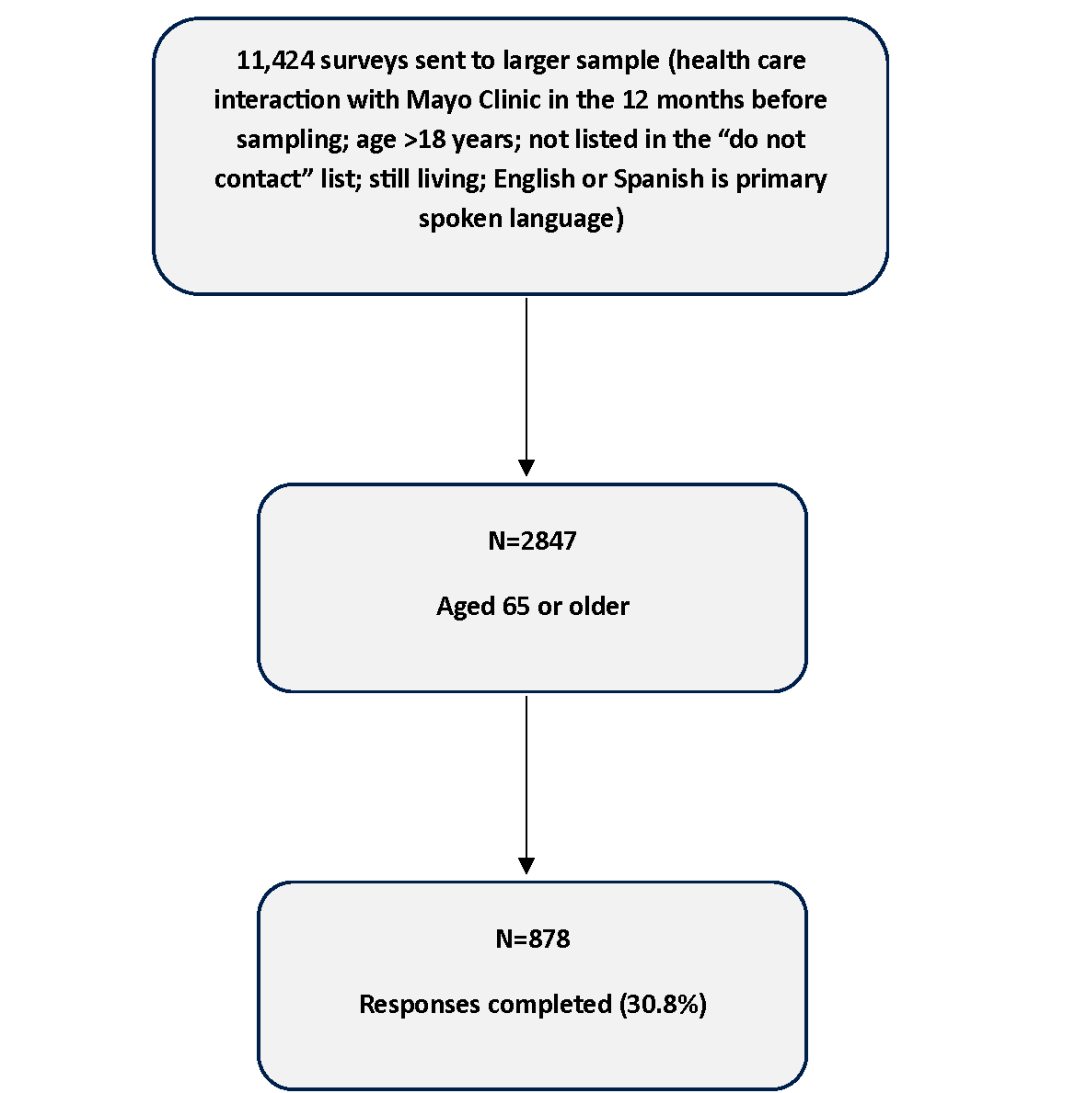
Flowchart of patients included in the survey sample.

An overview of the study inclusion is shown in Figure 1. A total of 2847 surveys were sent to patients aged ≥65 years. Of those, we received 878 complete responses (response rate 30.8%); 49.7% (436/878) of respondents completed the paper-based survey and 50.3% (442/878) of respondents completed the electronic survey. Survey respondents had a reported average age of 74.5 (SD 6.3) years; 51.3% (n=448) were women and 82.1% (n=715) were White. Across the entire response population, more than 81% (n= 711) of respondents had access to a computer or a smartphone and more than 93% (n=816) of respondents could access and connect to the internet to surf and use email (Table 1).

**Table 1.** Demographic characteristics of patients aged ≥65 years completing a survey on digital health literacy.

*Demographic characteristics	Participants (N=878), n (%)
Gender	
Missing	5 (0.6)
Women	448 (51.3)
Men	417 (47.8)
Transgender women	1 (0.1)
Prefer not to answer	5 (0.6)
Other	2 (0.2)
Race	
Missing	7 (0.8)
Asian/Pacific Islander	37 (4.2)
Black/African/African American	34 (3.9)
Mexican	9 (1.0)
Middle Eastern	2 (0.2)
Mixed	27 (3.1)
Native American/Native Hawaiian	16 (1.8)
No Answer	15 (1.7)
None/Other	16 (1.8)
White	715 (82.1)
Self-rated health	
Missing	7 (0.8)
Excellent	77 (8.8)
Very Good	287 (33)
Good	325 (37.3)
Fair	157 (18)
Poor	25 (2.9)
Comfort level: reading English	
Missing	9 (1.0)
Comfortable	848 (97.6)
Neutral	19 (2.2)
Uncomfortable	2 (0.2)
Comfort level: spoken English	
Missing	5 (0.6)
Comfortable	856 (98.1)
Neutral	16 (1.8)
Uncomfortable	1 (0.1)
Socioeconomic deprivation (Area Deprivation Index >=80%)	
Missing	78 (8.9)
Yes	49 (6.1)
No	751 (93.9)
Is patient’s mobility restricted?	
Missing	8 (0.9)
Yes	182 (20.9)
No	688 (79.1)
Connect to internet to surf web/email?	
Missing	20 (2.3)
Yes	802 (93.5)
No	52 (6.1)
I don’t know	4 (0.5)
Access internet from home?	
Missing	23 (2.6)
Yes	824 (96.4)
No	29 (3.4)
I don’t know	2 (0.2)
Satisfaction in ability to access internet?	
Missing	53 (6.0)
Satisfied	694 (84.1)
Neither	100 (12.1)
Dissatisfied	31 (3.8)
Device usage	
Desktop/Laptop Computer	
Yes	714 (81.3)
No	164 (18.7)
Tablet	
Yes	481 (54.8)
No	397 (45.2)
Smartphone	
Yes	747 (85.1)
No	131 (14.9)
None of the above	
Yes	12 (1.4)
No	866 (98.6)

### Self-Rated Health and Digital Health Literacy

Overall, most respondents felt confident in knowing what health resources were available, where, and how to find them, how to use the resources, and how to evaluate and use the information found in those resources (Table 2). However, less than half of the respondents felt confident in using and being able to determine the quality of health information found on the internet. While nearly 80% (n=673) of respondents could send a message to a doctor easily on the day, less than 45% (n=379) felt they could easily use a camera or video with a doctor on the day. Most respondents in each self-rated health group felt that they could send a message to a doctor easily on the day, though there were significant differences in distributions becoming more pronounced with fewer respondents reporting being able to do this among groups with lower self-rated health. In contrast, most (n=41, 54.7%) of the respondents in the excellent self-rated health group reported being able to use a camera or video with a doctor on the day, while this was the case for less than half of respondents with very good (n=132, 47.3%) and good (n=147, 46.4%) self-rated health, and lower for those in fair (n=54, 35.5%) and poor (n=5, 20.8%) self-rated health.

**Table 2.** Self-rated health by digital literacy among patients aged ≥65 years completing a survey on digital health literacy.

Self-rated digital health literacy variables	Excellent (n=77), n (%)	Very good (n=287), n (%)	Good (n=325), n (%)	Fair (n=157), n (%)	Poor (n=25), n (%)	Total (N=871), n (%)	*P* value
Health resources - What is available							.01
Yes	53 (71.6)	190 (68.1)	190 (60.1)	81 (52.9)	11 (45.8)	525 (62.1)	
No	7 (9.5)	45 (16.1)	63 (19.9)	39 (25.5)	5 (20.8)	159 (18.8)	
I don’t know	14 (18.9)	44 (15.8)	63 (19.9)	33 (21.6)	8 (33.3)	162 (19.1)	
Health resources - Where to find							.02
Yes	63 (84)	208 (74)	239 (74.7)	93 (61.2)	15 (62.5)	618 (72.5)	
No	4 (5.3)	36 (12.8)	37 (11.6)	28 (18.4)	5 (20.8)	110 (12.9)	
I don’t know	8 (10.7)	37 (13.2)	44 (13.8)	31 (20.4)	4 (16.7)	124 (14.6)	
Health resources - How to find them							.005
Yes	64 (86.5)	215 (77.1)	245 (77.3)	95 (62.5)	15 (62.5)	634 (74.9)	
No	4 (5.4)	34 (12.2)	36 (11.4)	28 (18.4)	5 (20.8)	107 (12.6)	
I don’t know	6 (8.1)	30 (10.8)	36 (11.4)	29 (19.1)	4 (16.7)	105 (12.4)	
Health resources - How to use							.02
Yes	62 (83.8)	221 (79.5)	238 (74.8)	97 (63.8)	15 (62.5)	633 (74.8)	
No	6 (8.1)	27 (9.7)	43 (13.5)	29 (19.1)	5 (20.8)	110 (13)	
I don’t know	6 (8.1)	30 (10.8)	37 (11.6)	26 (17.1)	4 (16.7)	103 (12.2)	
Health information - How to use							.02
Yes	54 (73)	214 (76.4)	223 (70.1)	95 (62.1)	15 (62.5)	601 (70.8)	
No	7 (9.5)	28 (10)	39 (12.3)	28 (18.3)	7 (29.2)	109 (12.8)	
I don’t know	13 (17.6)	38 (13.6)	56 (17.6)	30 (19.6)	2 (8.3)	139 (16.4)	
Health information - skills to evaluate							.003
Yes	58 (78.4)	166 (59.5)	186 (59.2)	78 (51)	10 (43.5)	498 (59.1)	
No	6 (8.1)	55 (19.7)	54 (17.2)	41 (26.8)	8 (34.8)	164 (19.5)	
I don’t know	10 (13.5)	58 (20.8)	74 (23.6)	34 (22.2)	5 (21.7)	181 (21.5)	
Health information - differentiate quality							.03
Yes	49 (66.2)	136 (48.9)	146 (46.2)	63 (41.2)	12 (50)	406 (48)	
No	9 (12.2)	70 (25.2)	72 (22.8)	41 (26.8)	8 (33.3)	200 (23.7)	
I don’t know	16 (21.6)	72 (25.9)	98 (31)	49 (32)	4 (16.7)	239 (28.3)	
Health information - confidence in using							<.001
Yes	49 (66.2)	122 (43.9)	135 (42.7)	46 (30.3)	10 (41.7)	362 (42.9)	
No	15 (20.3)	85 (30.6)	105 (33.2)	63 (41.4)	10 (41.7)	278 (32.9)	
I don’t know	10 (13.5)	71 (25.5)	76 (24.1)	43 (28.3)	4 (16.7)	204 (24.2)	
Able to send a message to doctor easily today?							<.001
Yes	68 (89.5)	233 (84.1)	260 (82.8)	99 (65.1)	13 (54.2)	673 (79.8)	
No	7 (9.2)	30 (10.8)	33 (10.5)	39 (25.7)	10 (41.7)	119 (14.1)	
I don’t know	1 (1.3)	14 (5.1)	21 (6.7)	14 (9.2)	1 (4.2)	51 (6)	
Able to use camera/video with doctor easily today?							.007
Yes	41 (54.7)	132 (47.3)	147 (46.4)	54 (35.5)	5 (20.8)	379 (44.7)	
No	29 (38.7)	121 (43.4)	148 (46.7)	77 (50.7)	18 (75)	393 (46.4)	
I don’t know	5 (6.7)	26 (9.3)	22 (6.9)	21 (13.8)	1 (4.2)	75 (8.9)	

### Mobility Restrictions and Digital Health Literacy

**Figure 2.**
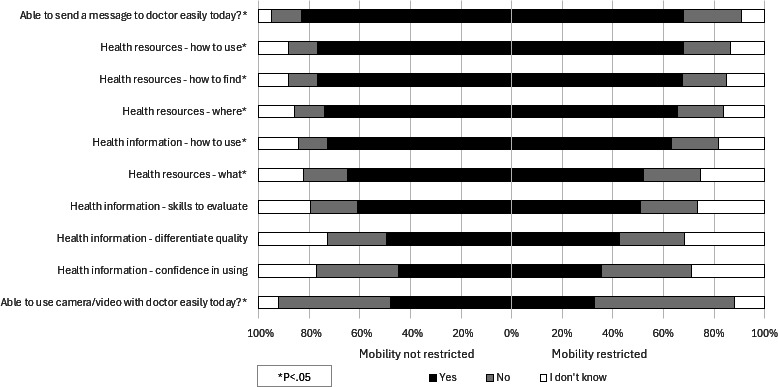
Mobility restrictions versus digital health literacy among older adults.

Respondents who reported restricted mobility were significantly different from those who reported no mobility restrictions in their ability to use digital health resources (Figure 2). They more often reported feeling unsure or not knowing how to use health resources online; however, they were more comfortable using health information found online. While most respondents felt they could send a message to a doctor, those reporting mobility restrictions differed significantly in their ability compared to those with no restricted mobility (n=121, 68.0% vs n=553, 83.2%). Most respondents were unsure or not able to use a camera or video with a doctor (n=468, 55.2%), and this was more often the case for those with mobility restrictions compared to those with no restricted mobility (n=120, 67.4% vs n=348, 52.0%). A significant difference in satisfaction with access to internet or a computer was observed with almost a quarter of respondents with mobility restriction being unsatisfied with their ability to access the internet (n=43, 23.5% vs n=94, 13.9%) or a computer (n=46, 25.3% vs n=114, 16.6%), compared to those with no restricted mobility.

### ADI and Digital Health Literacy

**Figure 3.**
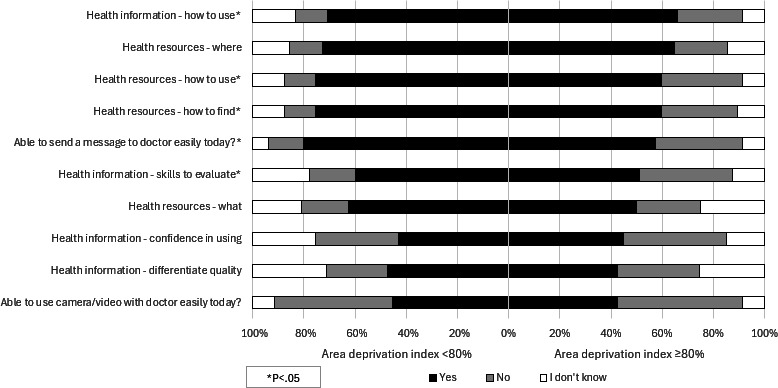
Social deprivation (measured by the Area Deprivation Index) by digital literacy among older adults.

When comparing respondents who experience socioeconomic deprivation as measured by ADI, a significantly higher proportion of those at or above 80% ADI reported not knowing how to find or use health resources and health information (Figure 3). Overall, 78.8% (n=611) of respondents were able to send a message to a doctor on the day; however, there was a significant difference between ADI groups with fewer of those at or above 80% ADI being able to do this compared with 45.3% (n=341) among those below 80% ADI. Less than half of the respondents overall were able to use a camera or video with a doctor; there was no significant difference between the ADI groups. There was a significant difference in access to a computer with 36.7% (n=18) of respondents at or above 80% ADI reporting no access to a computer versus 17.8% (n=134) among those below 80% ADI.

## Discussion

### Principal Results

In our survey among adults aged ≥65 years after the end of the COVID-19 pandemic, we successfully identified factors associated with older adults’ ability to access and use digital health resources. Our findings demonstrate that low self-rated health, mobility impairments, and socioeconomic disadvantages were associated with lower digital health literacy. Specifically, respondents with poor self-rated health showed substantially reduced ability to send messages to doctors (54.2% vs 89.5% with excellent health) and use video communication. Similarly, those with mobility restrictions showed lower confidence in finding and using health resources online and were less able to send messages to providers (68.0% vs 83.2% without restrictions). Respondents living in areas with high socioeconomic deprivation also showed significant disparities, mainly in messaging providers (57.4% vs 80.2% in low-deprivation areas) and access to computers (36.7% reporting no access vs 17.8% in low-deprivation areas).

### Interpretations, Implications, and Comparison With Previous Work

We observed significant relationships between low self-rated health and lower confidence and ability to use eHealth resources. This finding aligns with previous reporting that poor health is associated with lower digital health literacy and competence in the use of technology [4]. Our study also supports earlier research that seniors face more challenges in adopting innovative technologies compared with the general population [6,28]. Older adults who are suffering from health issues may have other care requirements taking precedence over the knowledge and use of digital resources, particularly when those resources are complicated, do not function as expected, or troubleshooting resources are not available.

A significant need exists to emphasize the importance of usability and accessibility in the design and development of digital health platforms [28,29]. Anxiety about the use of information and communication technologies, an issue disproportionately affecting seniors [30], can be reduced through encouragement and support of professionals, such as point-of-care teaching or advice by telephone. A patient portal eLearning program with older adults has been proven to increase self-efficacy and portal use among older adults [31] and could be tailored to specific subpopulations. Patients with poor self-rated health or suffering from cognitive decline may need shorter, more frequent sessions. Implementing a standardized assessment of patients’ digital readiness before scheduling telehealth visits would help identify those needing more support.

Our finding that less than 45% of responders felt confident using video with the provider suggests that previsit technical preparation is essential. Our data show that only 54.2% of individuals with poor self-rated health could message their provider, highlighting the need to establish a formal proxy user such as a family member or caregiver to help with digital health tasks while supporting privacy protections. Our finding that mobility limitations are associated with lower confidence and ability to use eHealth technologies aligns with earlier research. In a survey of 7609 community-dwelling Medicare beneficiaries, technology use decreased significantly with greater limitations in physical capacity and greater disability after adjustment for sociodemographic and health characteristics [12]. In a survey of 8019 individuals between the ages of 75 and 99, individuals with impaired functional ability or vision perceived digital health to be less beneficial than their counterparts with less impairment [29].

Older adults who are homebound and disabled due to mobility issues may have difficulties using nonadaptable devices. User interfaces requiring physical coordination (eg, camera installations or adjustments), dexterity (eg, holding a handheld device steadily), or sitting at a computer make these platforms less useful to individuals with physical limitations. Overcoming these obstacles requires addressing both the technical aspect of access with adaptable access options to people with age-related physical decline [32] and improving the functional aspect of access. Technology developers should prioritize user-centered designs such as larger text options, simplified navigation, sharp contrast displays, and voice command capabilities that can accommodate those with visual, cognitive, or dexterity limitations. Our findings that only 32.6% of mobility-restricted older adults felt comfortable using video with their doctor suggest that video interfaces require significant redesign for this population. Enhancing remote training capabilities to enable telemedicine visit engagement has been evaluated [33] and may provide opportunities to bridge the digital divide in the older adult subpopulation with mobility limitations.

Our finding that older adults living with socioeconomic deprivation report lower rates of knowledge of how to find or use eHealth resources and the ability to message their provider adds to extant literature. Previously published literature has proven that higher ADI correlates with lower telemedicine usage, particularly video-based consultations [8-11]. Compared with patients with a lower ADI in our survey, patients with high ADI were not more likely to report difficulty with engaging with video but did report more difficulty with sending messages to their doctor.

A digital divide has also been shown among patients with socioeconomic deprivation which varies by technology. Among patients living in economically disadvantaged zip codes, 32%‐40% of patients own a computer or an iPad compared to 59% from more affluent neighborhoods [34]. However, among individuals reporting a pretax annual income of < $35,000 per year, 96% report owning a smartphone, although only 15% report using a medical app [35]. Furthermore, consumer technology is being embraced by older adults (aged ≥65 y) [36] and the number of older adults who own a smartphone has risen from 18% in 2013 to 61% in 2021 among individuals aged ≥65 years [37]. In our survey, 85.1% of patients reported having a smartphone. Patients with socioeconomic deprivation based upon census data are more likely to access their medical records from a mobile device [38]. Facilitating care through mobile devices, templated instructions, and anticipatory guidance has been proposed as a practical solution to bridge the digital divide among patients with socioeconomic deprivation [39]. Given that 36.7% of respondents in high-deprivation areas reported no access to a computer, health care organizations should consider setting up technology lending programs that provide devices (tablets and laptops) and Internet connectivity to socioeconomically disadvantaged older patients. These programs could be integrated with existing social services and community outreach initiatives.

### Limitations and Strengths

Our study has several limitations. First, the cross-sectional design does not allow for assessing changes over time. Longitudinal studies are needed to understand how digital literacy evolves as health status, mobility, and socioeconomic circumstances change. Second, the use of self-reported data is limited by recall bias and may not accurately reflect participants’ actual digital capabilities. Third, we did not identify the specific comorbidities, functional limitations, or deprivation domains associated with lower use of eHealth resources. Fourth, surveys were sent to patients receiving care at a single health system, with most respondents being White and within a limited geographical area, which reduces generalizability to more diverse populations, those receiving care in different health care settings, or those living in different geographic regions with varying internet infrastructure. Analysis of nonresponse showed a significant difference in the distribution of race between responders and nonresponders, with higher proportions of non-White race groups among nonresponders. Finally, although our study was conducted after the acute phase of the COVID-19 pandemic, the extraordinary circumstances of the pandemic may have altered digital health usage in ways that will not persist in the future.

Our study has several strengths. First, we oversampled patients who were not registered with our patient portal to ensure representation from this group. Second, we assessed perceptions, intent, and use grounded in a well-known theoretical model (ie, technology acceptance model) which expands our understanding of actual uptake and use of eHealth solutions. Third, our use of validated assessment tools enhances the accuracy and applicability of the findings.

### Conclusions

Our findings highlight digital disparities of older adults associated with lower self-rated health, mobility limitations, and socioeconomic deprivation. These results have important implications for health care policy and practice and may allow health care systems to proactively identify older patients who would benefit from targeted interventions.

Rather than adopting a one-size-fits-all approach to digital health implementation, our data suggests the need for user-centered platforms with adaptable accessibility options that can accommodate the diverse needs of aging populations. This includes developing mobile-optimized solutions for socioeconomically disadvantaged patients who may lack computer access, implementing proxy user systems for those with poor health status, and redesigning video interfaces for mobility-restricted individuals.

Digital health tools present both an opportunity and a challenge: they can help close longstanding gaps in care or further marginalize those already facing barriers, especially older adults. Our findings underscore the importance of designing digital health systems with equity in mind.

## Supplementary material

10.2196/70672 Multimedia Appendix 1 Digital health inclusion survey.

10.2196/70672 Multimedia Appendix 2 Assessment for nonresponse bias.

## Abbreviations

ADI: Area Deprivation Index
EQ-5D: EuroQual-5 dimension scale
SED: socioeconomic deprivation
