# Supplementary material for: Assessing the Ability to Use eHealth Resources Among Older Adults: Cross-Sectional Survey Study
Source: JMIR Form Res. 2025 Aug 6;9:e70672. doi: 10.2196/70672 (PMC12327909; doi:10.2196/70672)
Supplement: Multimedia Appendix 2 [file formative-v9-e70672-s002.docx]

Supplemental Table 2. Analysis of Non-response

|  | | | | |
| --- | --- | --- | --- | --- |
|  | No response (N=1969) | Response (N=878) | Total (N=2847) | p value |
| **Age** |  |  |  | 0.0538 |
| N | 1969 | 878 | 2847 |  |
| Mean (SD) | 74.3 (7.2) | 74.5 (6.3) | 74.4 (6.9) |  |
| Median | 72.9 | 73.6 | 73.1 |  |
| Q1, Q3 | 68.4, 79.0 | 69.5, 78.4 | 68.8, 78.8 |  |
| Range | (65.0-105.0) | (65.0-97.7) | (65.0-105.0) |  |
|  |  |  |  |  |
| **Gender** |  |  |  | 0.7279 |
| Female | 1002 (50.9%) | 453 (51.6%) | 1455 (51.1%) |  |
| Male | 967 (49.1%) | 425 (48.4%) | 1392 (48.9%) |  |
|  |  |  |  |  |
| **Race** |  |  |  | <0.0001 |
| Asian | 150 (7.6%) | 47 (5.4%) | 197 (6.9%) |  |
| Black | 183 (9.3%) | 39 (4.4%) | 222 (7.8%) |  |
| Native American | 43 (2.2%) | 16 (1.8%) | 59 (2.1%) |  |
| Other | 119 (6.0%) | 38 (4.3%) | 157 (5.5%) |  |
| White | 1474 (74.9%) | 738 (84.1%) | 2212 (77.7%) |  |
|  | | | | |
